# Supplementary material for: Trends in tobacco, alcohol and branded fast-food imagery in Bollywood films, 1994-2013
Source: PLoS One. 2020 May 29;15(5):e0230050. doi: 10.1371/journal.pone.0230050 (PMC7259671; doi:10.1371/journal.pone.0230050)
Supplement: S1 File — (DOCX) [file pone.0230050.s001.docx]

**Supplementary File 1: Films included in analysis**

| **1994**  1942: A Love Story  Aatish  Anjaam  Dilwale  Dulara  Hum aapke hai koun  Insaniyat  Krativeer  Laadla  Main khiladi tu anari  Mohra  Raja babu  Suhaag  Vijaypath  Yeh dillagi | **1995**  Akele hum akele tum  Barsaat  Bombay  Coolie no. 1  Dilwale dulhaniya le jayenge  Jallad  Karan arjun  Nazayaaz  Raja  Ram jaane  Rangeela  Sabse bada khiladi  Trimurti  Yaraana  Haqeeqat | **1996**  Agani sakshi  Bandit queen  Darrar  Diljale  Ghatak  Hindustani  Jeet  Jung  Khamoshi; The musical  Khiladiyon ka khiladi  Krishna  Raja hindustani  Saajan chale sasural  Ajay  Sapoot | **1997**  Bhai  Border  Chachi 420  Diwana mastana  Dil to pagal hai  Gupt  Hero no. 1  Ishq  Judaai  Judwaa  Koyla  Pardes  Virasat  Yes boss  Ziddi | **1998**  Bade miyan chote miyan  Bandhan  China gate  Dil se  Dulhe raja  Dushman  Gulam  Jab pyar kisi se hota hai  Kuchh kuchh hota hai  Major saab  Pyar kiya to darna kya  Pyar to hona hi tha  Satya  Soldier  Zakhm | **1999**  Baadshah  Biwi no. 1  Daag  Haseena man jayegi  Hello brother  Hindustan ki kasam  Hum aapke dil me rehte hai  Hum dil de chuke sanam  Hum sath sath hai  Jaanwar  Khoobsurat  Mann  Refugee  Sarfarosh  Taal | **2000**  Badal  Dhadkan  Dulhan hum le jayenge  Fiza  Hadh kar di apne  Hamara dil aapake pass hai  Har dil jo pyar karega  Hera pheri  Josh  Kaho na pyar hai  Kya kehna  Mela  Mission Kashmir  Mohabbatein  Pukar |
| --- | --- | --- | --- | --- | --- | --- |
| **2001**  Ajnabee  Chandni bar  Chori chori chupke chupke  Dil chahta hai  Ek rishta  Farz  Gadar  Indian  Jodi no.1  Kabhi khushi kabhi gham  Kasoor  Lagaan  Mujhe kucch kehna hai  Style  Tum bin | **2002**  Aankhen  Yeh dil aashiqana  Awaara pagal deewana  Company  Deewangee  Devdas  Ek choti si love story  Hum tumhare hain sanam  Kaante  Kya yahi pyar hai  Maa tujhe salaam  Mere yaar ki shaadi hai  Raaz  Saathiya  Mujhse dosti karoge | **2003**  Andazz  Baghban  Bhoot  Chalte chalte  Hungama  Isaq vishq  Kal ho na ho  Koi mil gaya  LOC Kargil  Main prem ki diwani hoon  Munna bhai MBBS  Qavamat  Tere naam  The hero  Tujhe meri kasam | **2004**  Ab tak chappan  Aitraaz  Dhoom  Garv  Gayab  Hulchul  Hum tum  Julie  Khakee  Main hoo naa  Masti  Mujse shaddi karogi  Murder  Veer zara  Yuva | **2005**  Apharan  Bluffmaster  Bunty aur babli  Dus  Garam masala  Hanuman  Kaal  Kalyug  Kya kool hai hum  Maine pyar kyun kiya  Mangal pandey  No entry  Salaam namaste  Sarkaar  Waqt (the race against time**)** | **2006**  36 china town  Apna sapna money money  Bhagam bhaag  Dhoom 2  Don  Fanaa  Golmaal  Kabhi alvida na kahena  Krish  Lage Raho Munna Bhai  Malamaal weekly  Phir hera pheri  Rang de basanti  Taxi no 9211  Vivah | **2007**  Bhool bhulaiya  Chak de! India  Cheeni kum  Dhamaal  Guru  Hey baby  Jab we met  Metro  Namastey London  Om shanti om  Partner  Shootout at Lokhandwala  Ta ra rum pum  Taare zameen par  Welcome |
| **2008**  Bachna ae hasino  Bhootnath  Dostana  Fashion  Ghajini  Golmaal returns  Jane tu ya jane na  Jannat  Jodha akbar  Phook  Rab ne bana di jodi  Race  Rock on  Singh is king  Tashan | **2009**  3 idiots  Ajab prem ki ghazab kahani  All the best  Blue  De dana dan  Dev D  Kambakkht ishq  Kaminey  Love aaj kal  New York  Paa  Raaz-2  Rocket singh  Wake up sid  Wanted | **2010**  Anjana anjani  Atithi tum kab jaoge?  Badmash company  Band baja barat  Dabangg  Golmaal 3  Housefull  I hate luv story  Ishqiya  Kites  My name is Khan  Once upon a time in Mumbai  Peepli live  Raajneeti  Tees maar khan | **2011**  Body guard  Delhi belly  Don 2  Mere brother ki dulhan  Ra.one  Ready  Rockstar  Singham  The dirty picture  Yamla pagla dewaana  Zindagi na milegi dobara  Desi boyz  Double dhamaal  Murder 2  Tanu weds manu | **2012**  Agneepath  Barfi  Bol bachchan  Cocktail  Dabaang 2  Ek tha tiger  Housefull 2  Jab tak hai jaan  Khiladi 786  Omg oh my god!  Raaz 3  Rowdy rathore  Son of sardar  Student of the year  Talaash | **2013**  Aashiqui 2  Bhaag milkha bhaag  Chennai express  Dhoom 3  Grand masti  Krish 3  Once upon a time in Mumbai dobaara  R...rajkumar  Raanjhanaa  Race 2  Ram leela  Satyagraha  Shootout at Wadala  Special 26  Yeh jawaani hai deewani | |
