# Supplementary figures and images for: Trends in tobacco, alcohol and branded fast-food imagery in Bollywood films, 1994-2013
Source: PLoS One. 2020 May 29;15(5):e0230050. doi: 10.1371/journal.pone.0230050 (PMC7259671; doi:10.1371/journal.pone.0230050)

**Supplementary File 2: Mean film duration by year**

**
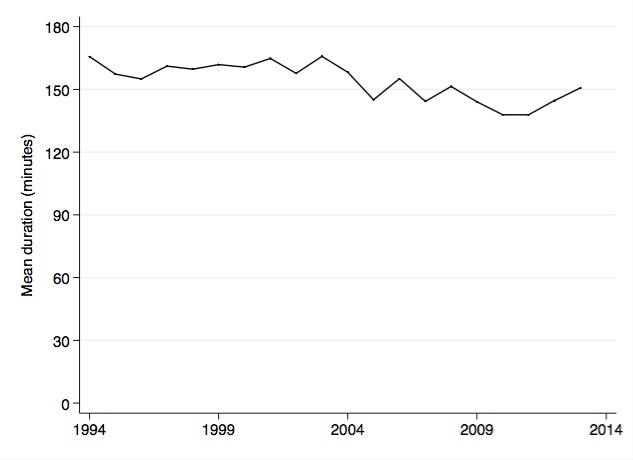
**

Supplement: S2 File — (DOCX) [file pone.0230050.s002.docx]

**Supplementary File 5: Distribution of film ratings, by year**


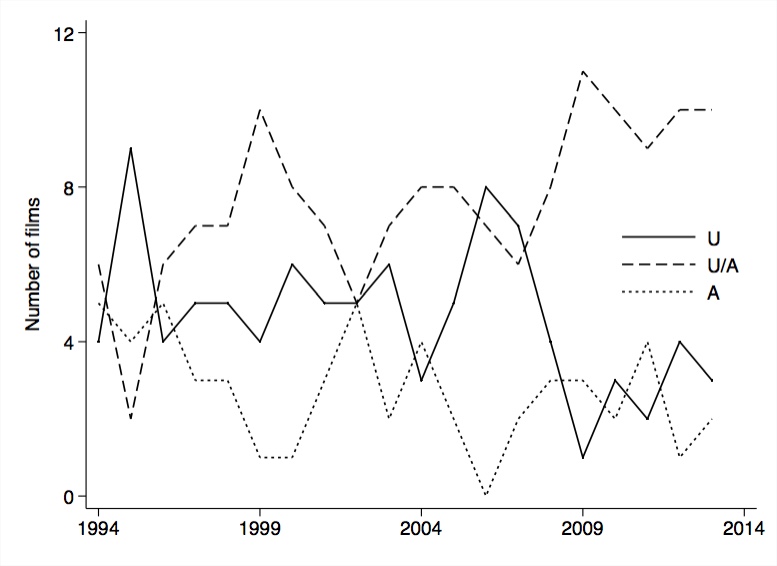

Supplement: S5 File — (DOCX) [file pone.0230050.s005.docx]
