# Supplementary material for: Trends in tobacco, alcohol and branded fast-food imagery in Bollywood films, 1994-2013
Source: PLoS One. 2020 May 29;15(5):e0230050. doi: 10.1371/journal.pone.0230050 (PMC7259671; doi:10.1371/journal.pone.0230050)
Supplement: S3 File — Proportion of films containing any tobacco, alcohol or fast-food image occurrence, and mean number of occurrences per film, by year. n = 15 films per year, 300 in total. (DOCX) [file pone.0230050.s003.docx]

**Supplementary File 3: Image occurrences by year**

Proportion of films containing any tobacco, alcohol or fast-food image occurrence, and mean number of occurrences per film, by year. n = 15 films per year, 300 in total.

|  |  | **Films with ≥1 image occurrence, n (%)** | | |  | **Mean occurrences per film ± SD** | | |
| --- | --- | --- | --- | --- | --- | --- | --- | --- |
|  |  | **Tobacco** | **Alcohol** | **Fast-food** |  | **Tobacco** | **Alcohol** | **Fast-food** |
| **Total** |  | 210 (70.0) | 278 (92.7) | 62 (20.7) |  | 4.0 ± 4.9 | 7.0 ± 4.7 | 0.4 ± 0.9 |
| **1994** |  | 14 (93.3) | 13 (86.7) | 0 |  | 7.1 ± 4.5 | 8.3 ± 6.2 | 0 |
| **1995** |  | 12 (80.0) | 14 (93.3) | 2 (13.3) |  | 6.3 ± 6.2 | 8.5 ± 4.6 | 0.3 ± 0.9 |
| **1996** |  | 13 (86.7) | 14 (93.3) | 0 |  | 4.4 ± 4.0 | 5.9 ± 3.8 | 0 |
| **1997** |  | 12 (80.0) | 15 (100.0) | 3 (20.0) |  | 4.3 ± 3.3 | 5.2 ± 2.9 | 0.3 ± 0.8 |
| **1998** |  | 12 (80.0) | 13 (86.7) | 2 (13.3) |  | 7.1 ± 6.7 | 5.3 ± 4.8 | 0.2 ± 0.6 |
| **1999** |  | 11 (73.3) | 13 (86.7) | 4 (26.7) |  | 3.4 ± 4.0 | 5.4 ± 4.2 | 0.3 ± 0.6 |
| **2000** |  | 12 (80.0) | 12 (80.0) | 2 (13.3) |  | 2.7 ± 2.3 | 5.0 ± 3.5 | 0.1 ± 0.4 |
| **2001** |  | 11 (73.3) | 15 (100.0) | 4 (26.7) |  | 3.9 ± 5.6 | 7.5 ± 4.6 | 0.4 ± 0.8 |
| **2002** |  | 12 (80.0) | 14 (93.3) | 2 (13.3) |  | 5.9 ± 6.6 | 5.9 ± 3.9 | 0.2 ± 0.6 |
| **2003** |  | 8 (53.3) | 12 (80.0) | 3 (20.0) |  | 3.0 ± 4.9 | 5.7 ± 4.8 | 0.3 ± 0.7 |
| **2004** |  | 13 (86.7) | 15 (100.0) | 2 (13.3) |  | 6.0 ± 5.7 | 6.9 ± 4.2 | 0.1 ± 0.4 |
| **2005** |  | 13 (86.7) | 14 (93.3) | 3 (20.0) |  | 4.4 ± 4.7 | 6.3 ± 2.9 | 0.4 ± 1.1 |
| **2006** |  | 7 (46.7) | 13 (86.7) | 5 (33.3) |  | 2.9 ± 5.0 | 6.2 ± 4.2 | 0.5 ± 0.9 |
| **2007** |  | 5 (33.3) | 13 (86.7) | 7 (46.7) |  | 1.9 ± 3.6 | 7.7 ± 4.7 | 1.0 ± 1.6 |
| **2008** |  | 9 (60.0) | 14 (93.3) | 4 (26.7) |  | 2.1 ± 4.2 | 7.3 ± 4.6 | 0.5 ± 0.9 |
| **2009** |  | 6 (40.0) | 15 (100.0) | 6 (40.0) |  | 2.5 ± 4.8 | 9.6 ± 4.7 | 0.9 ± 1.7 |
| **2010** |  | 8 (53.3) | 15 (100.0) | 4 (26.7) |  | 2.8 ± 4.6 | 8.9 ± 6.6 | 0.5 ± 1.1 |
| **2011** |  | 12 (80.0) | 15 (100.0) | 3 (20.0) |  | 3.7 ± 4.6 | 9.1 ± 4.4 | 0.5 ± 1.2 |
| **2012** |  | 12 (80.0) | 14 (93.3) | 3 (20.0) |  | 3.2 ± 2.9 | 7.2 ± 3.9 | 0.2 ± 0.4 |
| **2013** |  | 8 (53.3) | 15 (100.0) | 3 (20.0) |  | 2.6 ± 4.9 | 8.8 ± 7.2 | 0.3 ± 0.7 |
