# Supplementary material for: Trends in tobacco, alcohol and branded fast-food imagery in Bollywood films, 1994-2013
Source: PLoS One. 2020 May 29;15(5):e0230050. doi: 10.1371/journal.pone.0230050 (PMC7259671; doi:10.1371/journal.pone.0230050)

**Supplementary File 4: Occurrences of tobacco and alcohol subtypes per film, by year**

a. Mean number of occurrences per film for tobacco sub-types

b. Mean number of occurrences per film for alcohol sub-types

**a**

**b**


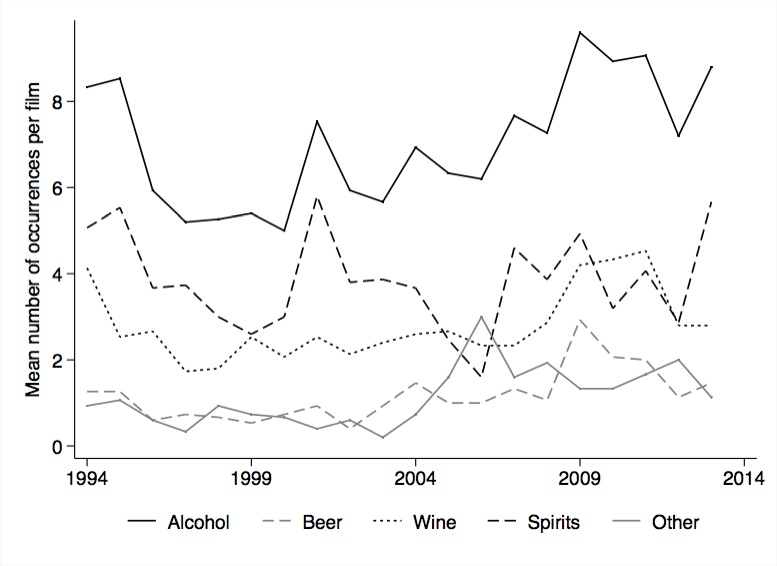

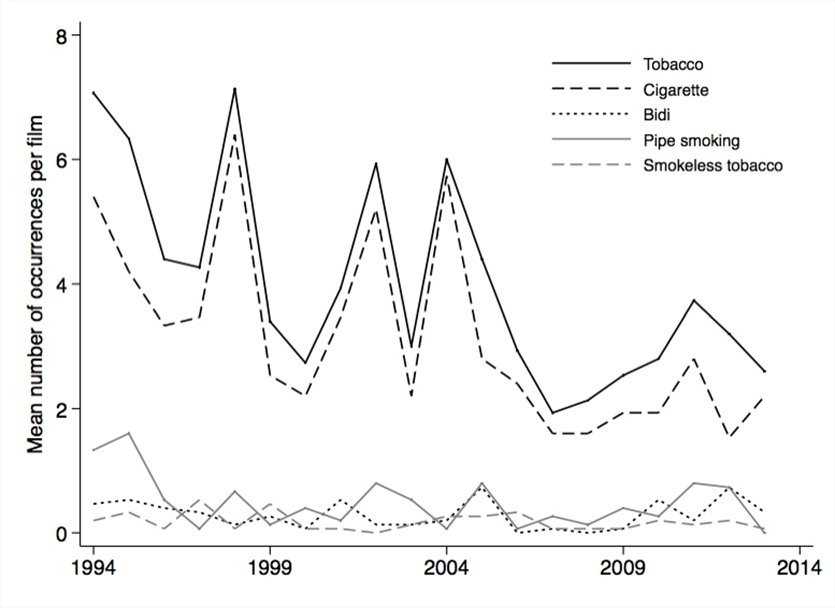

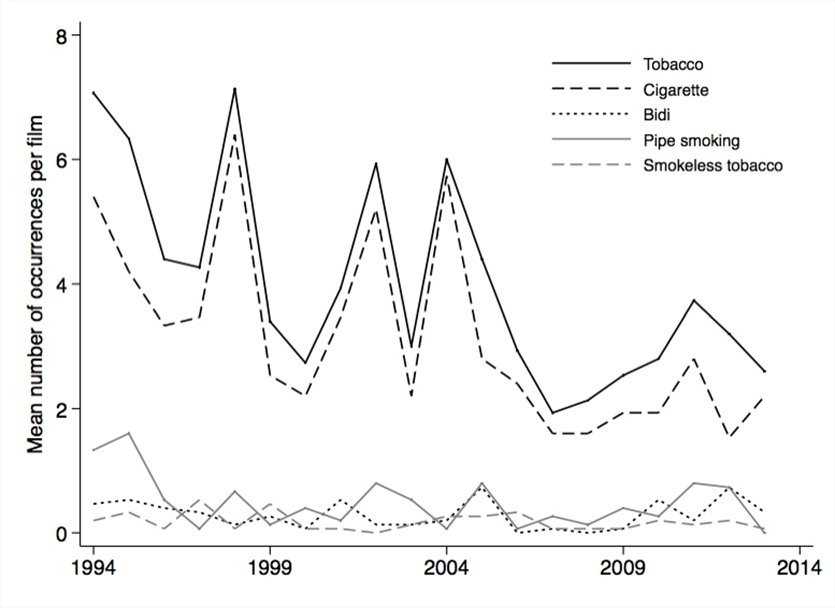

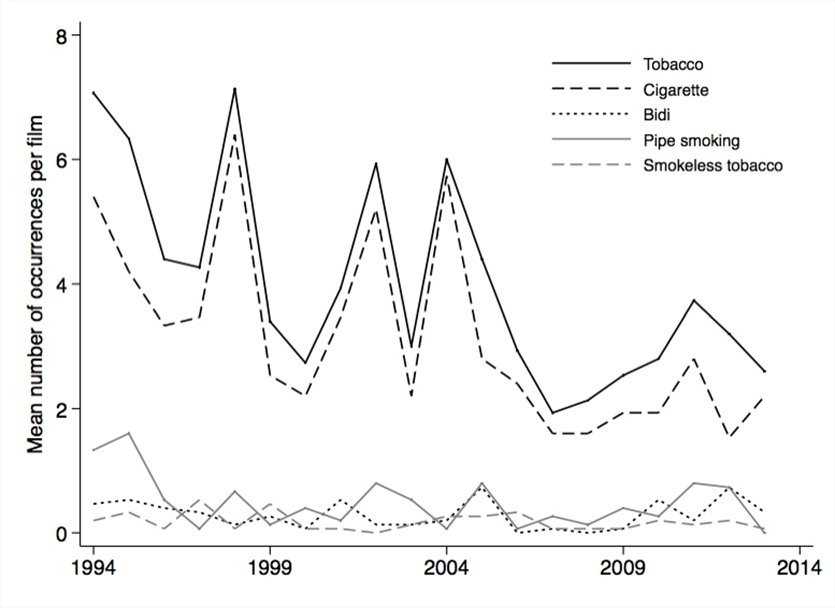

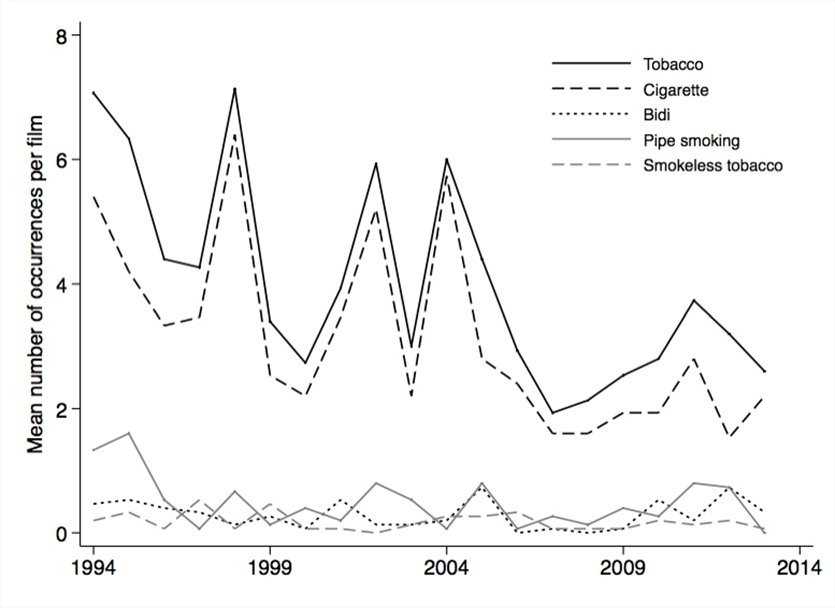

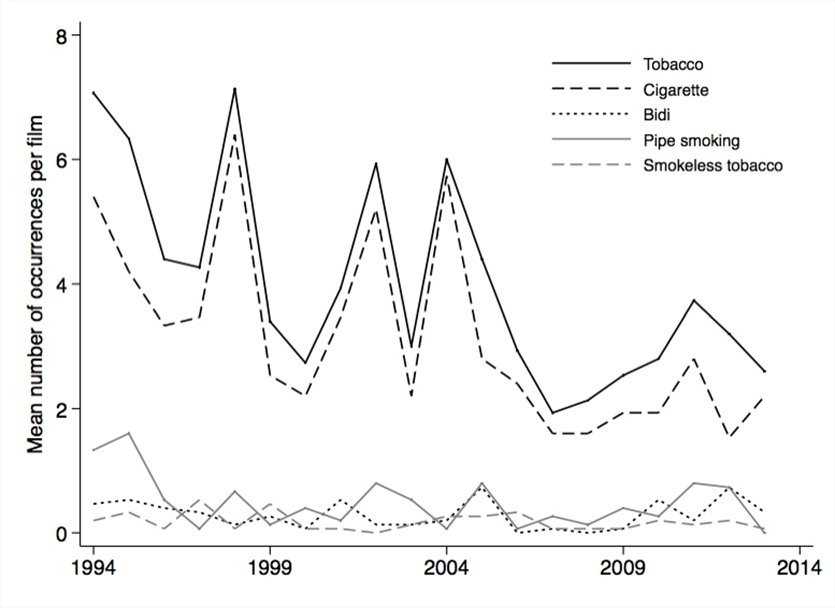

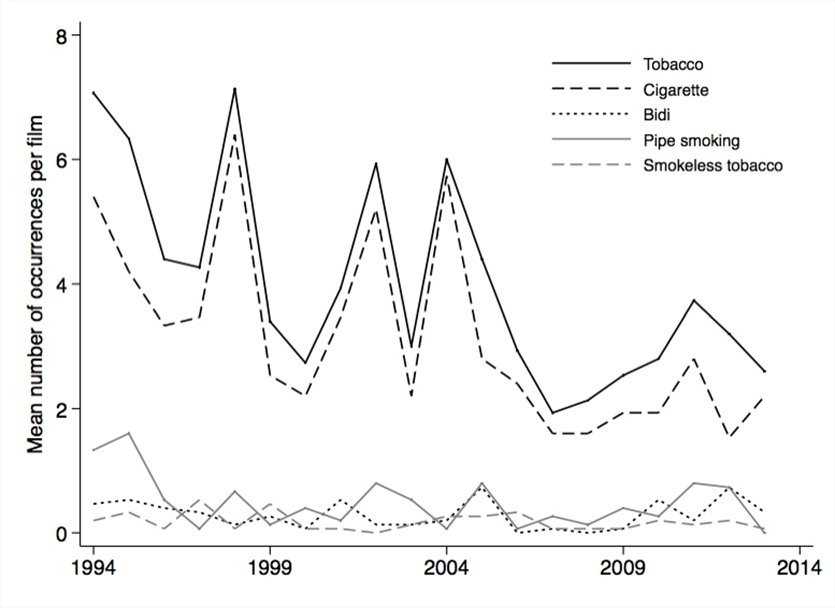

Supplement: S4 File — a. Mean number of occurrences per film for tobacco sub-types. b. Mean number of occurrences per film for alcohol sub-types. (DOCX) [file pone.0230050.s004.docx]
