# Supplementary material for: Trends in tobacco, alcohol and branded fast-food imagery in Bollywood films, 1994-2013
Source: PLoS One. 2020 May 29;15(5):e0230050. doi: 10.1371/journal.pone.0230050 (PMC7259671; doi:10.1371/journal.pone.0230050)
Supplement: S6 File — Mean number of tobacco (a), alcohol (b), and fast-food (c) occurrences/film per year, by rating category. Numbers of films per category per year (d). (DOCX) [file pone.0230050.s006.docx]

**Supplementary File 6: Tobacco, alcohol and fast-food occurrences/film per year, stratified by film rating**

Mean number of tobacco (a), alcohol (b), and fast-food (c) occurrences/film per year, by rating category. Numbers of films per category per year (d).


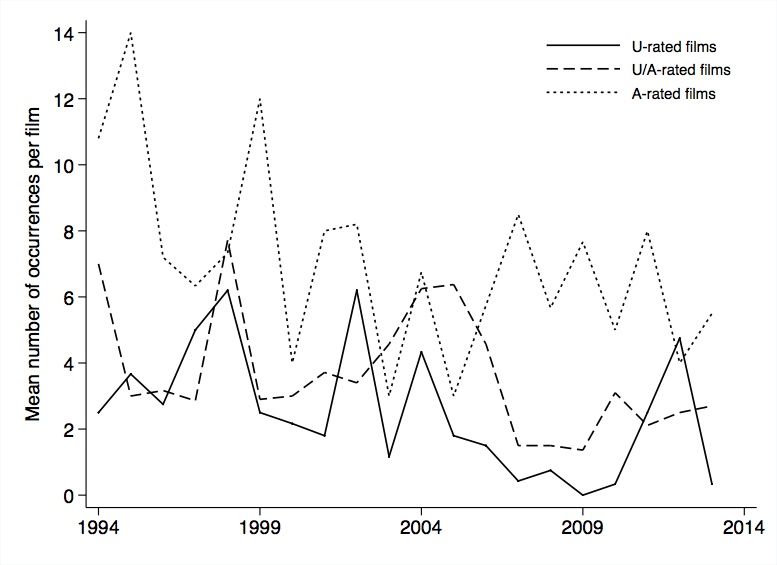


**a**


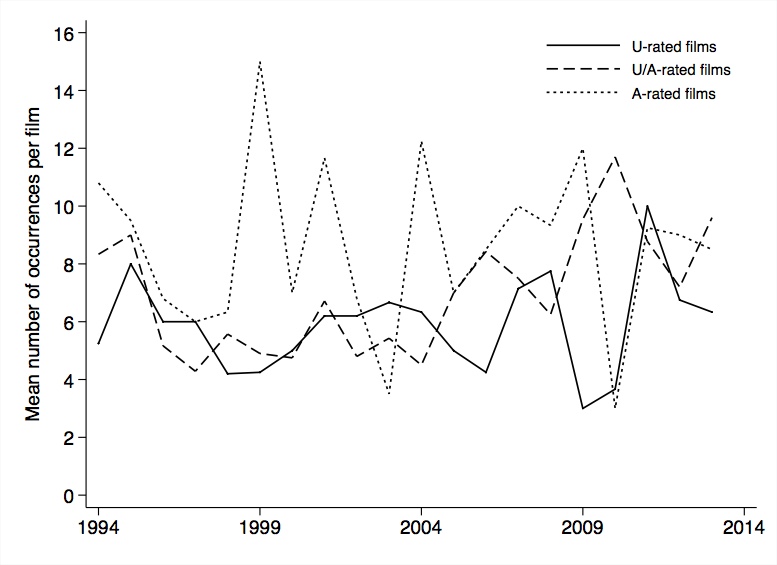


**b**


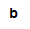


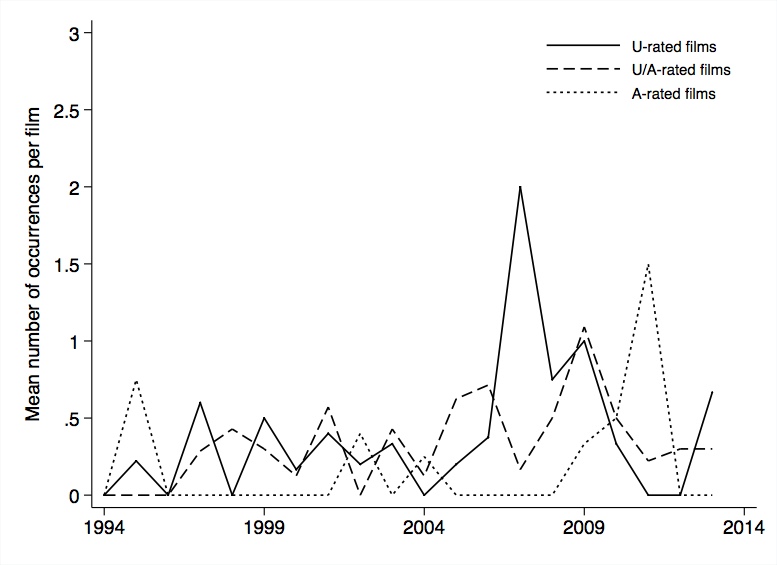


**c**

**d**

| **Film rating** | **Number of films (%)** | | | | | | | | | |
| --- | --- | --- | --- | --- | --- | --- | --- | --- | --- | --- |
|  | **1994** | **1995** | **1996** | **1997** | **1998** | **1999** | **2000** | **2001** | **2002** | **2003** |
| **U** | 4 | 9 | 4 | 5 | 5 | 4 | 6 | 5 | 5 | 6 |
| **U/A** | 6 | 2 | 6 | 7 | 7 | 10 | 8 | 7 | 5 | 7 |
| **A** | 5 | 4 | 5 | 3 | 3 | 1 | 1 | 3 | 5 | 2 |
|  | **2004** | **2005** | **2006** | **2007** | **2008** | **2009** | **2010** | **2011** | **2012** | **2013** |
| **U** | 3 | 5 | 8 | 7 | 4 | 1 | 3 | 2 | 4 | 3 |
| **U/A** | 8 | 8 | 7 | 6 | 8 | 11 | 10 | 9 | 10 | 10 |
| **A** | 4 | 2 | 0 | 2 | 3 | 3 | 2 | 4 | 1 | 2 |
